# Modulating Polyphenol Activity with Metal Ions: Insights into Dermatological Applications

**DOI:** 10.3390/pharmaceutics17020194

**Published:** 2025-02-04

**Authors:** Oana Cioanca, Ionut-Iulian Lungu, Denisa Batir-Marin, Andreea Lungu, George-Alexandru Marin, Riana Huzum, Alina Stefanache, Nazim Sekeroglu, Monica Hancianu

**Affiliations:** 1 Department of Pharmacognosy, Faculty of Pharmacy, “Grigore T. Popa” University of Medicine and Pharmacy, 700115 Iasi, Romania; 2 Department of General and Inorganic Chemistry, Faculty of Pharmacy, “Grigore. T. Popa” University of Medicine and Pharmacy, 700115 Iasi, Romania; 3 Department of Pharmaceutical Sciences, Faculty of Medicine and Pharmacy, Dunarea de Jos University, 800010 Galati, Romania; 4 Faculty of Medicine, “Grigore T. Popa” University of Medicine and Pharmacy, 400347 Iasi, Romania; 5 Faculty of Pharmacy, “Grigore T. Popa” University of Medicine and Pharmacy, 700115 Iasi, Romania; 6 Department of Food Engineering, Faculty of Engineering and Architecture, Kilis 7 Aralık University, 79000 Kilis, Turkey

**Keywords:** polyphenol–metal combinations, wound healing, antimicrobial, anticancer, nutraceuticals

## Abstract

Background: The skin represents the first barrier of defense, and its integrity is crucial for overall health. Skin wounds present a considerable risk seeing how their progression is rapid and sometimes they are caused by comorbidities like diabetes and venous diseases. Nutraceutical combinations like the ones between polyphenols and metal ions present considerable applications thanks to their increased bioavailability and their ability to modulate intrinsic molecular pathways. Methods: The research findings presented in this paper are based on a systematic review of the current literature with an emphasis on nanotechnology and regenerative medicine strategies that incorporate polyphenols and metallic nanoparticles (NPs). The key studies which described the action mechanisms, efficacy, and safety of these hybrid formulations were reviewed. Results: Nanocomposites of polyphenol and metal promote healing by activating signaling pathways such as PI3K/Akt and ERK1/2, which in turn improve fibroblast migration and proliferation. Nanoparticles of silver and copper have antibacterial, angiogenesis-promoting, inflammation-modulating capabilities. With their ability to induce apoptosis and restrict cell growth, these composites have the potential to cure skin malignancies in addition to facilitating wound healing. Conclusions: Nanocomposites of polyphenols and metals provide hope for the treatment of cancer and chronic wounds. Their antimicrobial capabilities, capacity to modulate inflammatory responses, and enhancement of fibroblast activity all point to their medicinal potential. Furthermore, these composites have the ability to decrease inflammation associated with tumors while simultaneously inducing cell death in cancer cells. Clarifying their mechanisms, guaranteeing stability, and enhancing effective delivery techniques for clinical usage should be the focus of future studies.

## 1. Introduction

One of the most difficult and widespread problems facing modern healthcare is the treatment of chronic wounds, which may take more than a month to heal. Wounds like this do not heal well for a variety of reasons; some of them include diabetes, venous insufficiency, and pressure ulcers. Effective care of chronic wounds involves tackling both the wound and the systemic factors that impede healing. To fully comprehend these wounds, a thorough knowledge of their genesis and pathophysiology is necessary. Healthcare resources are being overwhelmed by the growing number of chronic wounds caused by obesity and an aging population. These wounds have a profound effect on patients’ quality of life and call for new ways of treating them to improve their healing results [1,2].

The yearly cost of treating and maintaining chronic wounds is in the billions of dollars, which results in huge economic expenses within healthcare systems worldwide; these wounds have severe physical, mental, and social effects, which negatively impact the patient’s quality of life. The need for effective care is shown by the fact that current figures indicate that 6.5 million people in the US alone suffer from chronic wounds [3]. A chronic wound is one that does not heal normally, meaning it does not undergo haemostasis, inflammation, proliferation, or remodeling. An unusual inflammatory response, characterized by the overproduction of pro-inflammatory cytokines, bacterial colonization, and poor angiogenesis, is often associated with these abnormal wounds; the treatment problem is worsened because this inflammation-damage spiral slows healing, which causes additional necrosis [4,5].

Fruits, vegetables, and herbs are rich sources of polyphenols, a varied family of phytochemicals known for their anti-inflammatory, antibacterial, and powerful antioxidant capabilities. With an expected increase in demand for both recreational and medicinal uses, the worldwide polyphenols market was worth over USD 1.67 billion in 2023 and will reach USD 2.4 billion in 2027. Flavonoids, phenolic acids, polyphenolic amides, and other types of polyphenols are present in variable amounts in different foods. Certain foods have high concentrations of these chemicals, such as berries, tea, red wine, and dark chocolate. Recent research suggests that polyphenols may considerably improve the healing process, expanding their medicinal potential beyond conventional uses. Acutely problematic in chronic wound settings due to the overproduction of reactive oxygen species (ROS), they exercise their actions via regulating inflammatory responses, increasing angiogenesis, and decreasing oxidative stress [6,7,8].

**Figure 1:**
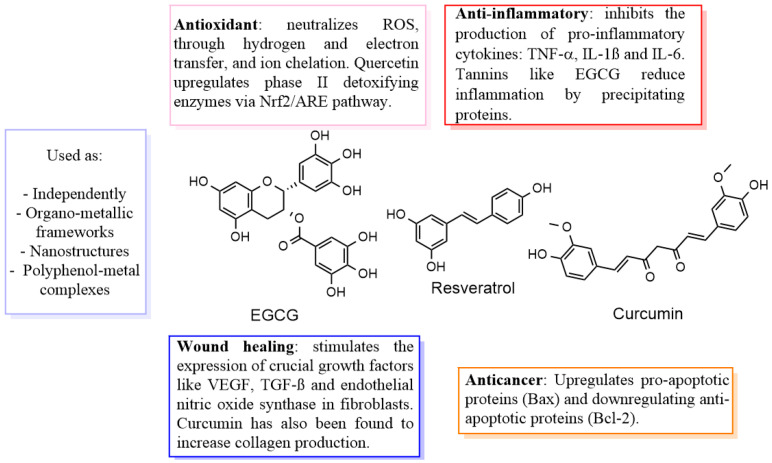
The diverse properties of polyphenols, ranging from anti-inflammatory to anticancer.

Polyphenols like flavonoids, for instance curcumin, have been found to suppress oxidative damage by acting as free radical scavengers and enhancing the body’s natural antioxidants including superoxide dismutase and catalase. In addition to this, curcumin has also been found to accelerate collagen production, an important biomolecule in the wound healing and especially scarring process, helping close open injuries and reduce bacterial infections. This is very important in the case of chronic wounds since one of the features is inflammation, which leads to the production of ROS. In this way, polyphenols help to reduce oxidative stress not only as a protective mechanism for the cells, but also as a more favorable environment for tissue healing (Figure 1) [9,10,11].

Nanomedicine and smart dressings are two examples of emerging technologies that could improve wound healing, infection prevention, and inflammation management. In order to better understand how these novel tactics work, how they are applicable in clinical practice, and what kind of research is still needed to enhance the treatment of chronic wounds, this study will analyze the existing literature on the topic [12,13].

The unique physical and chemical properties of nanoscale materials provide an opportunity for the creation of innovative drug delivery systems, the improvement of antimicrobial capabilities, and the acceleration of wound healing; because nanotechnology can precisely deliver medications to the injured region with little or no side effects and high concentrations, it has found extensive usage in wound care [14].

This study will focus on flavonoids, phytoalexins, catechins, and polyphenol-rich plant extracts as means to synthesize nanoparticles, for their use in dermatology, for afflictions like chronic wounds, pressure ulcers, skin infections, and skin cancer.

## 2. Literature Review Process

A comprehensive literature review was performed for this article, using international public sources including Google Scholar, PubMed, and ScienceDirect, to obtain the necessary data. Keywords like “polyphenol-metal complexes”, “wound-healing”, “topical application”, “skin cancer”, “dermal infection”, and “nutraceuticals” were used to identify relevant papers for our investigation.

**Figure 2:**
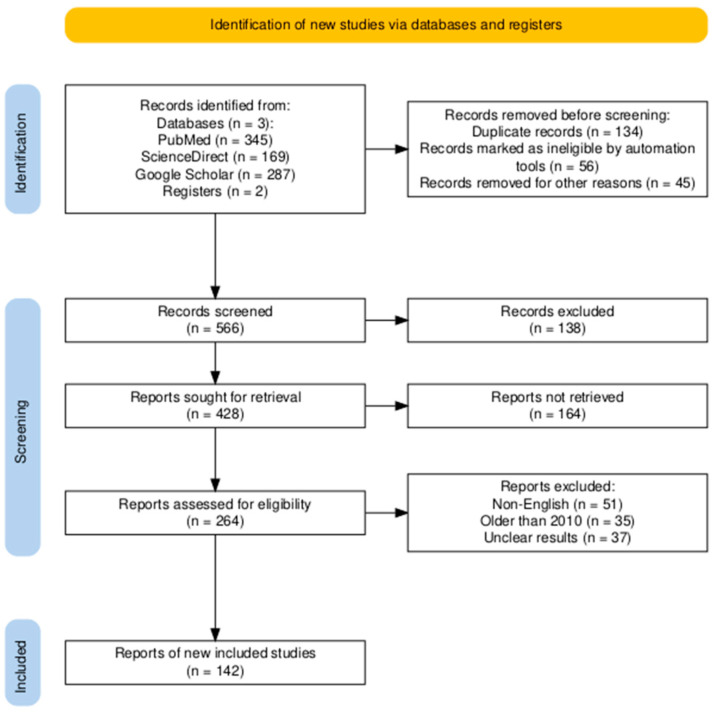
The selection process designed for this review’s subject to extract only pertinent material.

Subsequent to this search, publications underwent a two-step screening procedure, as seen in Figure 2, made using protocols designed by Haddaway et al. (2022) [15]. The articles were selected based on the information provided in the title and abstract. The year of publication was significant, selecting only publications published post-2010, with exceptions for important works.

Secondly, the publications underwent a more stringent filtration process, resulting in the exclusion of non-peer-reviewed studies and those lacking succinct information about the effects of polyphenols and metal complexes in dermal applications. Ultimately, all publications remaining following this procedure were included in a citation database.

The data extraction focused on essential facts about the attributes of various kinds of polyphenol–metal nanoparticles and complexes and their proven effects in wound-healing, inflammation, skin infections, and cancer.

This review was designed to discuss a particular problem, that being the use of green-synthesized nanoparticles and complexes for topical and transdermal use. Seeing that not many studies of this kind are present in the current literature, this work will contribute greatly to our understanding of nutraceuticals in dermatological care.

## 3. Wound Healing Aided by Polyphenol–Metal Combinations

Wound healing is a complex biochemical process that aims at restoring the structure of the tissue to its normal state after an injury; it consists of several distinct and consecutive stages: haemostasis, inflammation, proliferation, and remodeling. All these stages are important; haemostasis is the body’s primary response to injury, which includes coagulation to ensure that there is no bleeding; inflammation is to remove debris and pathogens from the wounded area. The proliferation phase is the tissue building and the re-epithelialization process which results in the formation of granulation tissue that is replaced by collagen during the remodeling phase. Delayed healing wounds or chronic wounds are classified as those which fail to heal within the expected time frame of four to six weeks and are associated with comorbidities leading to sustained inflammation and dysregulated tissue responses. Chronic diseases not only inhibit healing but also increase the risk of infection and make the recovery even more challenging [16,17,18,19].

Polyphenols’ properties are enhanced when they are conjugated with metal NPs to produce composite nanomaterials with enhanced therapeutic effects, presented in Table 1. Silver NPs are known to possess excellent antibacterial effects as they are capable of disrupting the microbial cell membrane and preventing biofilm formation, which are the two most important problems in the management of chronic wounds. The synergy between polyphenols, such as quercetin, and AgNPs leads to enhanced fibroblast migration and proliferation through the activation of key signaling pathways, including the PI3K/Akt and ERK1/2 pathways, which are critical for collagen synthesis and tissue repair. Additionally, polyphenols modulate inflammation by inhibiting the NF-κB pathway, thereby reducing levels of pro-inflammatory cytokines like IL-1β and TNF-α, essential for maintaining a controlled inflammatory response at the wound site. The presence of AgNPs not only aids in preventing biofilm formation—one of the primary challenges in chronic wounds—but also promotes angiogenesis by upregulating vascular endothelial growth factor (VEGF) and fibroblast growth factor (FGF), ultimately facilitating neovascularization [20,21,22,23].

In addition to silver, other metal NPs like copper are also being used due to their physiological functions that are vital in wound healing. Copper is an essential element that is involved in numerous enzymatic processes that include collagen formation and angiogenesis [31,32]. Copper has also been demonstrated to enhance the expression of VEGF signaling pathways, which results in the formation of new blood vessels and the healing of injured tissue. When copper NPs are conjugated with polyphenols such as resveratrol, composites are formed and the biological activity of these composites is enhanced with the ability to heal wounds and the factors that cause chronic wounds [33,34,35,36].

The use of polyphenol–metal composites in the formulation of hydrogel is another latest and rather smart approach through which these bioactive compounds can be deposited at the wound site and at the same time, provide the right moist environment that is required for healing. The non-bioactive hydrogels can be compatible with polyphenol–metal NPs to provide a constant and local delivery of the therapeutic agents. The hydrogels are able to cover the wound and can also change with the wound condition since they are dynamic; they can change in response to pH and temperature to enhance the therapeutic effects. For example, hydrogels that are sensitive to inflammation will release more polyphenols when there are pro-inflammatory cytokines present, thereby providing a way of targeting the wound’s specific needs [37,38,39,40].

**Figure 3:**
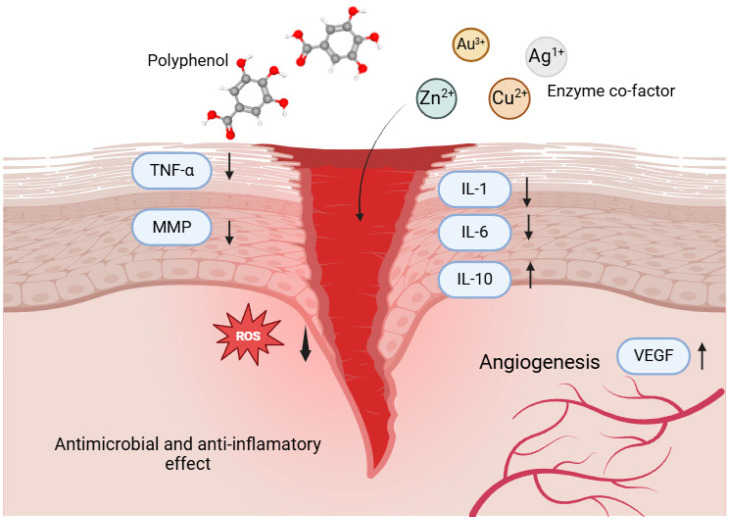
The biochemical effects of polyphenol–metal combinations on wound healing.

Tang et al. (2023) extensively discussed the integration of metallic elements with herbal compounds in microneedle applications, emphasizing their potential to enhance wound healing processes. Microneedles act as minimally invasive delivery systems, facilitating direct deposition of polyphenol–metal complexes into the dermal layers, promoting localized therapy [41]. For instance, zinc, known for its regulatory role in collagen synthesis and its ability to stabilize antioxidant enzymes, worked synergistically when complexed with polyphenols to expedite tissue regeneration and reduce healing time. Zinc’s contribution to the metabolism of key growth factors involved in the wound healing cascade further supported this synergistic approach (Figure 3) [42,43,44,45].

Johnson et al. (2022) researched the pervasive challenge of oxidative stress in chronic wounds; oxidative stress fosters a hostile wound environment, prolonging inflammation and delaying healing. Polyphenols can mitigate this as potent free radical scavengers via their oxidative stress-mitigating capabilities, when complexed with metals like copper, known not only for its essential role in angiogenesis and skin reconstruction but also for its ability to modulate pro-inflammatory cytokines [46]. The synergistic effect of the polyphenols and metals could be further enhanced through precise formulations, offering a targeted approach to disrupt chronic inflammation [47,48].

The application of NPs is a focal point in advancing wound healing technologies. Huang et al. (2024) introduced polyphenol-based photothermal NPs, which exhibit a unique ability to modulate the wound microenvironment by reducing the production of ROS and inflammatory cytokines, while at the same time, stimulating the formation of new blood vessels and oxygenating the wound. These NPs leverage near-infrared light to generate heat, aiding in microbial eradication while simultaneously promoting blood flow, a crucial factor in accelerating healing in diabetic wounds. This heat-induced modality stimulated cellular activities crucial for repair, including fibroblast proliferation and angiogenesis; the incorporation of polyphenols within these NPs not only enhanced their thermal stability but also maintained their biological activities, showcasing the combination’s multimodal approach to wound management [49]. According to the work conducted by Blanco-Fernandez et al. (2021), copper NPs hold the capacity of promoting wound healing through the promotion of endothelial cell growth and new blood vessel formation [25].

The stability and bioavailability of these complexes are critical factors in their application; Ohanyan et al. (2024) detailed the use of tannin–albumin particles as stable carriers for therapeutic agents, which have significant potential in ensuring longevity and controlled release; the stability provided by albumin in these complexes allowed for sustained release, maintaining therapeutic levels longer than conventional formulations [50]. This stability is particularly vital for delivering consistent therapeutic doses over prolonged periods, thus reducing the frequency of applications and improving patient compliance; their findings align with previous studies highlighting the advantages of biopolymer-based carriers in drug delivery formulated for targeted applications [51,52].

Nakajima et al. (2023) delved into the engineering of bioactive nanocomplexes that possessed targeted delivery capabilities, enhancing therapeutic precision in gingival and potentially dermal applications. This innovative work showcased not only the utility of polyphenols and metals but also their potential for application in tissue regeneration beyond dermal contexts. The targeted delivery mechanisms in their study underscored the importance of localizing treatment to reduce systemic side effects while ensuring effective drug action at the wound site [53].

Polyphenols are known for their anti-inflammatory properties and thus have the potential of being used as wound healing enhancers; the prevention of over inflammation, which can worsen the condition of the wound and delay the healing process, also requires control of cytokines that mediate inflammation such as IL-1 β and TNF-α [54,55,56,57]. These cytokines are needed for the inflammatory response. According to Gowda et al. (2023), polyphenols can decrease cytokine levels and thus, lead to a decrease in chronic wound inflammation. With the appearance of polyphenol–metal composites, there is a new strategy in the treatment of infections as these compounds also have anti-inflammatory effects [58].

Even if these novel materials hold great promise for use in the clinical setting, there are certain issues that have to be considered before they can be implemented in practice. However, their potential to tackle the barriers to healing, including infection and inflammation, offers hope to transform the management of wounds. The use of polyphenolic compounds that modulate several cellular pathways in conjunction with the antimicrobial effects of metal NPs provides a basis for the creation of advanced wound dressings that are adaptable to the dynamic nature of chronic wounds.

## 4. Polyphenol–Metal Combinations Against Bacteria

Bacterial killing is as a result of the damage that is caused to the cell membranes of the microorganisms; hence, they die due to the lack of oxygen as well as the release of ROS. This action can be further coupled with an unattractive environment for the bacteria as polyphenols can modulate inflammatory response and target bacterial metabolism. This stemmer, as pointed out by Zhang et al. in 2024, improves the healing time through a reduction in inflammation and the control of infection [59].

Building up the therapeutic applications of polyphenol–metal mixtures, the use of copper NPs and complexes along with polyphenols, for instance, resveratrol [60] or quercetin [61,62,63] and gallic acid [64,65], has demonstrated quite encouraging results in combating bacterial and fungal infections.

The use of metals in polyphenols provides an interesting perspective on how these two act. The formation of ROS is an important mechanism through which metal NPs exhibit antimicrobial activity. Some of the effects include damage on bacterial DNA, lipid peroxidation, and cell death through oxidative stress. This method is especially useful in the fight against biofilm forming bacteria which are very hard to eradicate as they have a protective extracellular matrix. It has been seen that bacteria are already in a compromised state as far as growth and survival is concerned and the addition of ROS further worsens the situation by enhancing cell stress and suppressing metabolic processes [66,67,68,69,70,71].

In research by Li et al. (2022), green-synthesis results in the formation of NPs that still possess the biological activity of the parent polyphenols and at the same time, is compliant with the principles of sustainable materials science [72].

The efficacies of the curcumin-conjugated silver NPs for the treatment of the antibiotic resistant bacterium, methicillin-resistant *Staphylococcus aureus* (MRSA), were demonstrated; research proved that these NPs cut down the numbers of bacteria in the infected wounds, which means that the polyphenol–metal hybrids might be new efficient antibacterial agents that could replace traditional antibiotics which are becoming less efficient due to bacterial resistance [73]. The former provides the antimicrobial effect to the mixture while at the same time, it enhances the structural integrity of the NPs and boosts their antibacterial activity. A common hypothesis is that this symbiosis is because of curcumin’s ability to interact with metal ions, thus altering the release profile and enhancing bioavailability at the site of infection [74,75,76,77].

Li et al. (2021) further explored innovations in wound care through the use of polyphenol and Cu^2+^-modified chitin sponges, which demonstrate remarkable antibacterial properties due to the intrinsic characteristics of both components. This study emphasized not only the direct antimicrobial action of these complexes but also their antioxidant capabilities, showing that layered treatment can target various aspects of the wound healing process [78]. The enhanced vascularization observed in their study suggests a profound impact on early wound closure and reduced scarring, attributed to copper’s facilitative role in VEGF signaling pathways and its ability to stimulate new capillary formation [79,80,81].

Table 2 provides a brief overview of recent polyphenol–metal combinations with proven antimicrobial activity.

Because of their complex action mechanisms, polyphenol–metal combos are very efficient against bacterial infections. The formation of ROS is an important fundamental process; silver and copper NPs stand out. When these nanostructures come into contact with bacterial cells, they have the ability to trigger reactions that result in increased levels of ROS [67,69,94,95]. Indirect cellular damage to DNA, proteins, and lipids results from this oxidative burst, which in turn, makes the environment unsuitable for bacterial life. Mohammadi et al. (2021) showed that silver NPs stabilized with curcumin greatly boosted ROS generation, leading to elevated oxidative stress and, eventually, the lysis and death of bacterial cells [96].

These composites have antibacterial properties because they induce oxidative stress and break bacterial membranes. For example, Siriphap et al. (2022) showed that cellular function and integrity are compromised due to changes in membrane permeability caused by EGCG incorporation in gold NPs [97]. When polyphenol–metal composites come into contact with bacterial membranes, they may cause changes in conformation that increase the membrane’s permeability and make it easier for metal ions and polyphenols to penetrate the cell. This action has two effects: first, it damages cells more effectively, and second, it blocks essential metabolic pathways that bacteria need to multiply [98,99,100,101,102].

Additionally, metal ions and polyphenols interact intricately, leading to a synergistic increase in antibacterial efficacy. The antibacterial and bioavailable effects of polyphenols may be enhanced by their complexation with metal ions. In a study by Rohatgi et al. (2023), for instance, antibacterial activity against *Pseudomonas aeruginosa* was shown to be improved when quercetin and copper NPs were used together. This was mainly because the copper ions generate ROS, while resveratrol modulates inflammatory pathways. In addition to influencing the bacteria themselves, this combination helped lower the inflammatory response [90].

Lastly, these combinations play a crucial role in modulating the host immunological responses. In order to reduce the excessive inflammation that often occurs with infections, polyphenols are able to control pro-inflammatory cytokines. According to Braga et al. (2019), quercetin–silver NPs have antibacterial properties against *Listeria monocytogenes* and significantly reduce cellular inflammation. Improving antibacterial efficacy and creating a healing environment are two goals of these composites, which target the infection and the inflammatory response via signal management [92].

## 5. Polyphenol–Metal Combinations as Future Anticancer Agents

Skin cancer, including melanoma, and other forms of skin cancer, such as basal cell carcinoma and squamous cell carcinoma, is a major health challenge across the globe. The etiology of skin cancer is diverse, and it involves UV radiation exposure, hereditary factors, and immunodeficiency mechanisms. Standard therapies include surgery, chemotherapy, and radiotherapy and can be successful; however, they are associated with numerous adverse effects and rely, to some extent, on the stage of the cancer. Due to the increasing demand for new and more effective skin cancer treatment options, researchers are working on the synthesis and use of polyphenol–metal hybrids as potential therapies for skin cancers [103,104,105,106,107].

The ways through which polyphenol–metal combinations display their anticancer potential are numerous and interrelated (Table 3), and they affect many cellular processes and signaling pathways.

The first mechanism is the induction of apoptosis or programmed cell death through which cancer cells are eliminated; silver NPs, especially the ones that are functionalized with curcumin, have been found to induce apoptosis in melanoma and other skin cancer cells effectively. The contact between the NPs and the cancer cells contributes to the production of ROS, which in turn, cause oxidative stress and apoptosis signaling [108,109,110,111].

**Table 3:** Molecular pathways and effects of polyphenol combinations on cancerous cells.

Combination	Effects	Molecular Pathway	References
Curcumin-Silver NPs	Induction of apoptosis; significant reduction in cell viability	Generation of ROS; activation of caspase pathways; inhibition of NF-κB signaling	[112,113,114]
Grape polyphenols + Gold NPs	Reduction in metastatic potential	Disruption of membrane integrity; modulation of MAPK pathways; induction of apoptosis via increased ROS	[115,116,117]
Resveratrol + Gold NPs	Inhibition of tumor growth; antiangiogenic effects	Activation of p38 MAPK pathway; suppression of VEGF; enhancement of ROS production leading to cell death	[118,119,120]
Quercetin + Zinc NPs	Inhibition of proliferation; enhancement of apoptosis	Inhibition of CDK activity leading to G1 arrest; reduction in pro-inflammatory cytokines	[121,122]

The superoxide anion is known to activate a number of pro-apoptotic factors like Bax while at the same time inactivating the antiapoptotic factors like Bcl-2. Such imbalances result in the release of cytochrome c from mitochondria, hence enhancing the activity of caspases that are well-known mediators of apoptosis [123].

Research showed that ROS levels are increased when curcumin-stabilized silver NPs are used and this results in the apoptosis of melanoma cells through the activation of apoptotic pathways [112].

**Figure 4:**
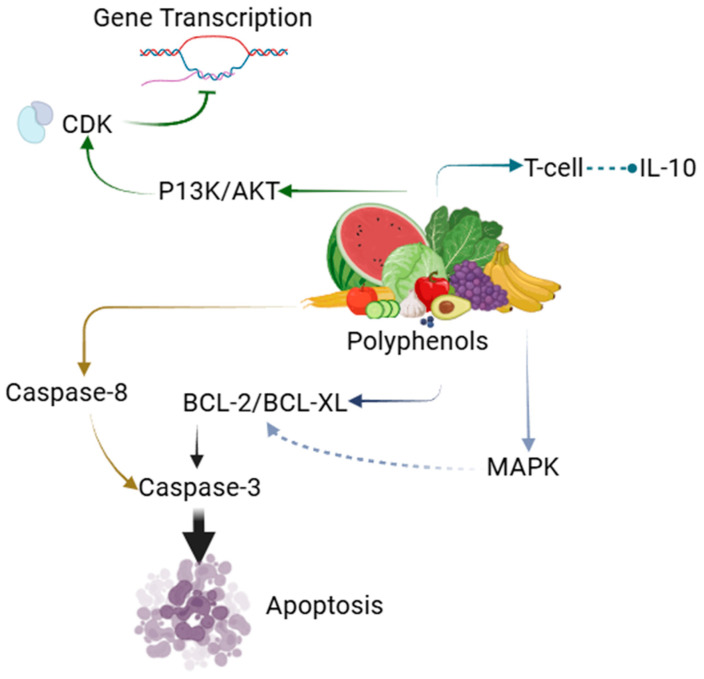
Molecular mechanisms through which polyphenols induce apoptosis in cancer cells.

Another important way through which polyphenol–metal combinations are thought to exhibit their anticancer potential is through the modulation of inflammatory pathways (Figure 4). Inflammation has been postulated to play a role in carcinogenesis and tumor growth; the anti-inflammatory properties of polyphenols make them act on the inflammatory environment that is associated with tumors. For instance, NF-κB, a transcription factor that works in the activation of inflammatory cytokines, is known to be suppressed by resveratrol when used together with copper NPs. Resveratrol also interferes with the expression of pro-inflammatory cytokines such as IL-1β and TNF-α, thus decreasing the inflammatory response and promoting tissue healing [124,125,126].

The polyphenols and metal NPs’ interaction does not only increase the effectiveness of the individual anticancer properties but also the ability of suppressing cell proliferation and metastasis. Polyphenols have been found to interfere with cell cycle regulation signaling pathways, which include the CDKs. For instance, CDK activity has been halted by curcumin, thus leading to cell cycle arrest at the G1 phase and preventing the proliferation of cancer cells. This activity is particularly helpful in the case of skin cancers since uncontrolled cell division is a key feature of the disease [127,128,129].

Also, the use of a polyphenol–metal combination may help in the enhancement of the apoptotic process through the modulation of these other signaling pathways. Notably, the MAPK pathway, which is a mitogen-activated protein kinase that governs several cellular activities including proliferation, differentiation, and apoptosis, is frequently altered in cancer. Some polyphenols have been also found to be able to activate the pro-apoptotic signals and to block the antiapoptotic ones through the MAPK cascade. For instance, curcumin has been proven to activate the p38 MAPK pathway in numerous cancer cell lines.When combined with silver or gold NPs, the overall pro-apoptotic signals could be stronger and, therefore, more effective against skin cancers [130,131,132,133].

In the work of Vladu et al. (2022), the authors show that the stability and the therapeutic effect of polyphenols are significantly increased when these are used together with metals such as zinc or copper and applied to skin tumors. For example, polyphenols combined with copper 2+ ions dissociate in biological liquids into the polyphenoxyl radical + and copper 1+; further, copper 1+ ions yield a Fenton reaction with intrinsically produced hydrogen peroxide to produce copper 2+ and hydroxyl anions, which will induce apoptosis in cancer cells. Some of the polyphenols, resveratrol, including curcumin, not only offer antioxidant and anti-inflammatory properties that are useful in the maintenance of skin health but also offer promising anticancer potential against different skin cancers, including melanoma and squamous cell carcinoma. When these compounds are delivered through transdermal delivery systems, these ingredients are able to penetrate through the skin to a certain extent to treat the tumors without affecting other parts of the body to a large extent. The use of metals in these formulations can help with the delivery and uptake of the formulations. For instance, the use of zinc in the formulation of curcumin has been seen to improve the bioavailability of the compound and its capacity to bring about apoptosis in skin cancer, thus improving the therapeutic effect [129].

The polyphenol–metal complexes’ antitumor activities are associated with a broad spectrum of the phenotypic modulation of the pathways critical to skin cancer development. Korkina et al. (2009) explain that the combination of polyphenols with metal ions can greatly increase the polyphenols’ ability to regulate the activity of NF-κB and MAPK, which are very important in cell survival and proliferation. For example, Cu^2+^–resveratrol has been found to be very effective in inducing apoptosis in melanoma cells through the activation of the AIF pathway [134]. Also, it has been established that when quercetin is used together with zinc, it can inhibit the activity of matrix metalloproteinases (MMPs), which are involved in skin cancer invasion and metastasis. These mechanisms explain how these combinations can be effective in delivering therapy that can interfere with the development of skin cancer and encourage the repair of normal tissue at the same time [135,136,137].

Current research also focuses on the use of topical as well as transdermal techniques for the enhancement of bioavailability as well as the therapeutic potential of polyphenol–metal mixtures. Leena et al. (2020) explain the benefits of using nutraceuticals in the form of topical preparations for the management of skin cancer. For instance, the copper–curcumin-loaded nanocarrier formulations can significantly increase the skin penetration of the drug through the stratum corneum and achieve a high drug concentration at the tumor site. Such systems exploit nanotechnology to encapsulate the complexes, enhancing targeted delivery that not only enhances the efficacy against skin tumors but also reduces the toxicity on normal skin cells. This is consistent with the current trends of developing and applying personalized medicine where the effects of certain treatments on individuals could lead to the development of specific topical gels. Also, these compounds have antioxidant properties that can be beneficial in protecting healthy skin during the concomitant therapies with radiation or chemotherapy, and thus improve the patients’ quality of life [138]. With the advancement of knowledge and the application of a polyphenol–metal combination in the formulation of topical gels, we can be in a position to change the face of skin cancer management, thus increasing the therapeutic indices and patient satisfaction.

The development of polyphenol–metal composites is not limited to their cytotoxic effects; these combinations also hold the promise of enhancing the overall therapeutic landscape for skin tumors through improved delivery systems and reduced side effects. The application of nanoencapsulation methods for the creation of stable formulations that deliver the drugs to the tumor site only ensures that the side effects are minimized, and that drug absorption is increased. For instance, the NPs can be developed in a way that polyphenols will be released in a gradual manner so as to maintain the therapeutic concentrations at the tumor site while minimizing the exposure all through the body.

Also, the efficacy of polyphenol–metal combinations for the inhibition of angiogenesis is of increasing interest for the comprehensive treatment of tumors. Angiogenesis is the formation of new blood vessels and is an essential process in the growth and spread of tumors. This way, polyphenol–metal composites can starve the tumors of the nutrients and oxygen that are needed for their survival by suppressing angiogenesis [137,138].

## 6. Conclusions

Chronic wounds are one of the major concerns in present-day healthcare, defined as wounds that take more than 12 weeks to heal. These wounds, which result from diseases including diabetes and pressure ulcers, not only have a negative impact on the patients’ quality of life but also are costly to healthcare systems. According to the estimation, 6.5 million people suffer from chronic wounds in the United States only; therefore, efficient management techniques are crucial to tackle this problem. This is because obstacles that hinder normal wound healing such as inflammation, microbial colonization, and angiogenesis pose a challenge to treatment and, therefore, require new approaches.

Through well-defined molecular mechanisms, polyphenol–metal nanocomposites show promising therapeutic benefits in the treatment of wounds and cancer. The PI3K/Akt and ERK1/2 signaling pathways are critical for collagen production and tissue regeneration; when combined with AgNPs, polyphenols like quercetin improve fibroblast migration and proliferation, which aids in wound healing. A regulated inflammatory response at the wound site may be maintained with the aid of these polyphenols, which modify inflammation by blocking the NF-κB pathway and lowering pro-inflammatory cytokines like IL-1β and TNF-α. Crucial for the management of chronic wounds, silver nanoparticles’ antibacterial characteristics break microbial membranes and limit biofilm development. When used to treat cancer, polyphenol–metal combinations, such silver nanoparticles functionalized with curcumin, promote cell death in cancer cells by releasing cytochrome c, which activates caspases, and by activating mitochondrial pathways and producing ROS. Curcumin promotes programmed cell death by increasing ROS generation and decreasing the levels of antiapoptotic proteins such as Bcl-2. In addition, these composites stop cancer cells from proliferating by suppressing NF-κB, which in turn, reduces inflammation associated with tumors and blocks cell cycle regulators including CDK. By interacting with various signaling pathways, polyphenols and metal ions have the potential to improve healing processes and target cancer.

Further research should be aimed at determining the mechanisms of action of these hybrids in the body, especially as they relate to modulating cellular and inflammatory responses. It is also important to establish the stability, bioavailability, and the proper delivery of these composites so that these outcomes can be achieved in the real world.

## Figures and Tables

**Table 1:** Polyphenol–metal composites reported in the literature for wound healing.

Combination	Properties	Mechanisms	Reference
Silver NPs + Curcumin	Enhances fibroblast activity	Scavenges ROS; enhances cell migration and proliferation	[24]
Copper NPs + Resveratrol	Essential for angiogenesis, promotes collagen synthesis	Stimulates endothelial cell proliferation; supports VEGF signaling for new blood vessel formation	[25,26]
Quercetin-Stabilized silver NPs	Provides dual antibacterial and anti-inflammatory benefits	Scavenges free radicals; modulates inflammatory pathways; enhances fibroblast activity	[22,27,28]
Polyphenol-Infused hydrogels	Biocompatible; maintains moist environment	Sustained release of bioactive compounds; modulates inflammation; facilitates localized delivery	[29,30]

**Table 2:** Polyphenol–metal combinations with proven antimicrobial activity.

Combination	Target Bacteria	Effects	References
Curcumin-Stabilized silver NPs	MRSA	Significant reduction in viability; inhibition of biofilm formation	[82,83,84,85]
Epigallocatechin gallate (EGCG) + Gold NPs	*Escherichia coli*	Broad-spectrum antibacterial activity; enhanced stability	[86,87,88]
Quercetin + Copper NPs	*Pseudomonas aeruginosa*	Increase in bioavailability; enhanced anti-inflammatory effects; biofilm inhibition	[89,90]
Quercetin + Zinc/Silver NPs	*Listeria monocytogenes*	Significant bacterial inhibition; membrane disfunction	[14,91,92,93]

## Data Availability

No new data were created.

## Abbreviations

The following abbreviations are used in this manuscript:

NPs	nanoparticles
US	United States
ROS	reactive oxygen species
VEGF	vascular endothelial growth factor
IL	interleukin
TNF-α	tumor necrosis factor alpha
DNA	deoxyribonucleic acid
EGCG	epigallocatechin gallate
MRSA	methicillin-resistant *Staphylococcus aureus*
UV	ultraviolet
CDK	cyclin-dependent kinases
MAPK	mitogen-activated protein kinase
MMPs	matrix metalloproteinase
